# Harnessing salt slag and diatomite sludge by co-recycling for zeolite production

**DOI:** 10.1038/s41598-026-50164-3

**Published:** 2026-04-24

**Authors:** Rafael Carrizosa, Isabel Padilla, Maximina Romero, Aurora López-Delgado

**Affiliations:** MEDES Group, Materials Department, Eduardo Torroja Institute for Construction Sciences, IETcc-CSIC. C/ Serrano Galvache, 4, 28033 Madrid, Spain

**Keywords:** Co-recycling, Salt slag, Diatomite sludge, LTA zeolite, NaP zeolite, Waste harnessing, Energy science and technology, Engineering, Environmental sciences, Materials science

## Abstract

Developing alternative uses for waste is one of the main concerns of modern industry, as proper waste management is necessary to pave the way towards a circular economy and sustainability. In this study, the sustainable synthesis of zeolites using salt slag, a hazardous waste product from the secondary aluminium industry, and diatomite sludge, an agri-food waste product, was explored. The aluminium salt slag and diatomite sludge served as the aluminium and silicon sources, respectively, for the LTA and NaP zeolite formulations through a one-pot hydrothermal process. The key synthesis parameters, temperature (70–90 °C), reaction time (2–24 h), and NaOH concentration (0.38–1 M), were systematically varied to adjust the type, crystallinity and textural properties of the zeolites. The synthesised materials exhibited specific surface areas of 12.7–22.5 m^2^ g^−1^ and cation exchange capacities ranging from 1.57 to 2.54 meq g^−1^. FTIR analysis confirmed the formation of zeolitic phases, whereas microstructural characterisation revealed a progressive topotactic transformation from cubic LTA to NaP crystalline aggregates. These findings demonstrate the potential of this approach to produce functional zeolites and promote circular economy practices by co-recycling industrial waste through industrial symbiosis.

**Supplementary Information:**

The online version contains supplementary material available at 10.1038/s41598-026-50164-3.

## Introduction

Aluminium is one of the most extensively recycled metals owing to its favourable physical and chemical properties and its critical role in strategic sectors such as transportation, construction, packaging, and renewable energy technologies. Global demand for aluminium is projected to increase by more than 50% by 2050, reaching approximately 107.8 million tonnes^1^. Despite the advantages of recycling, aluminium production remains highly energy-intensive, consuming 3–5% of global electricity and contributing approximately 3% of worldwide greenhouse gas emissions^2^. Addressing this growing demand requires robust strategies that reinforce circular economy principles within the aluminium sector, not only by reducing energy consumption and emissions, but also by managing the significant volumes of waste generated during recycling processes^3,4^.

Secondary aluminium production, based on the reprocessing of scrap, offers clear advantages over primary production, including lower costs and substantially reduced energy consumption. As a result, the share of secondary aluminium in global output has steadily increased^5^. Within this sector, fluxing salts are commonly used during melting to enhance metal recovery from heterogeneous scrap streams. This practice leads to the generation of salt slag, one of the most problematic waste streams associated with secondary aluminium production. On average, approximately 0.5 tonnes of salt slag are generated per tonne of aluminium recovered^6,7^. Given that more than 26 million metric tonnes of recycled aluminium are expected to be produced worldwide by 2027^8^, salt slag generation may reach approximately 13 million metric tonnes annually.

The chemical and mineralogical composition of salt slag is highly variable, depending on scrap composition and recycling conditions, and typically includes metallic aluminium, metal oxides, chlorides, and minor compounds such as nitrides, fluorides, carbides, sulphides, and phosphides^7,9^. Salt slag is classified as hazardous waste under the European Waste Catalogue (EWC code 10 03 08*)^10^ and is characterised by high flammability, irritancy, harmfulness, and leachability. Improper disposal may result in soil and water contamination by toxic metal leachates, as well as the release of hazardous gases (NH_3_, CH_4_, H_2_, PH_3_, and H_2_S) upon contact with moisture^11^. Consequently, the large-scale generation and hazardous nature of salt slag represent a global environmental challenge^12^.

Current salt slag treatment processes generally involve mechanical separation to recover metallic aluminium and salt, followed by leaching of the fine fraction to dissolve residual salts^12–16^. The resulting non-metallic residue is typically valorised in the construction sector^5,17–19^. An alternative and increasingly explored approach is the utilisation of salt slag as an aluminium source for zeolite synthesis^20,21^.

Zeolites are crystalline microporous aluminosilicates widely used in the chemical industry due to their high reactivity, selectivity, and structural versatility. Their three-dimensional frameworks contain well-defined channels and cavities that confer outstanding catalytic, adsorptive, and ion–exchange properties^22^. The global zeolite market (natural and synthetic) is expected to reach approximately USD 14 billion by 2026, with synthetic zeolites accounting for nearly USD 6 billion in 2023 and dominating applications in detergents and adsorbents^23^. In this context, transforming salt slag into zeolite feedstock represents an attractive route to valorise a hazardous waste while generating high-value materials.

Zeolite synthesis from salt slag requires the addition of a suitable silicon source to adjust the Si/Al ratio. In this work, diatomite sludge from the brewing industry was selected as an alternative silica-rich waste material. Diatomite, also known as diatomaceous earth, is a fine sedimentary rock of biogenic origin that is mainly composed of amorphous silica (SiO_2_) derived from the fossilised siliceous frustules of diatoms^24^. It is widely used as a filtration aid, particularly in beer production^25,26^. After use, spent diatomite is commonly discarded as sludge and classified as waste under EWC code 02 07 05^10^.

The co-recycling of salt slag and diatomite sludge therefore represents a promising strategy for simultaneously valorising two industrial wastes. Among synthetic zeolites, Linde Type A (LTA, zeolite A) is one of the most commercially relevant, accounting for approximately 73% of total synthetic zeolite production due to its extensive use as a detergent builder^27,28^. Its high cation exchange capacity enables efficient removal of calcium and magnesium ions from water. Zeolite NaP, belonging to the GIS topology, also exhibits high ion-exchange capacity and is particularly effective in binding calcium ions due to its narrower pore system^29–31^. Both zeolites are widely applied in water softening, wastewater treatment, and environmentally friendly detergent formulations^32–36^.

Interest in producing zeolites from non-conventional, waste-derived raw materials has increased substantially in recent years. Waste streams rich in silicon and aluminium offer sustainable alternatives to conventional precursors and can yield zeolites with competitive performance in metal removal and gas or organic pollutant adsorption^37–39^. With respect to diatomite, zeolites have been synthesised mainly from natural diatoms^40,41^, but the use of diatomite waste for this purpose has rarely been explored^42^. Although several studies have reported the synthesis of LTA, NaP, SOD, and ANA zeolites from aluminium wastes using commercial sodium silicate solutions^43,44^, and others have employed extracted silicate or aluminate solutions from waste materials^21^, the direct use of two different waste streams in a single synthesis step remains scarcely explored^44^. To the best of the authors´ knowledge, no one-pot process has been reported that directly converts aluminium salt slag and diatomite sludge into high-quality zeolites without intermediate extraction or purification steps.

Accordingly, the objective of this study is to demonstrate the feasibility of recycling hazardous aluminium salt slag and diatomite sludge through a one-pot hydrothermal synthesis of zeolites. The influence of synthesis parameters on phase formation and material properties is systematically investigated, and the potential of this approach as a sustainable co-recycling strategy aligned with industrial symbiosis and circular economy principles is evaluated.

## Materials and methods

### Raw materials

Aluminium salt slag (AlSS) was supplied by Alusigma, S.A., a secondary aluminium smelter located in Asturias, Spain. This waste is collected from the furnace after the heat treatment of aluminium scrap in the production of aluminium ingots. It is a greyish solid waste with an unpleasant odour and a total aluminium content, expressed as Al_2_O_3_ of 63.5 wt%, distributed among different mineralogical phases, including metallic aluminium, spinel (Al_2_MgO_4_), and corundum (Al_2_O_3_). Other components include 7.9 wt% MgO, 7.7 wt% SiO_2_, and 4.5 wt% CaO, together with minor metal oxides and salts. Details of the chemical and mineralogical composition of AlSS, as well as its treatment and hydrolysis, have been described in a previous study^20^.

**Fig. 1:**
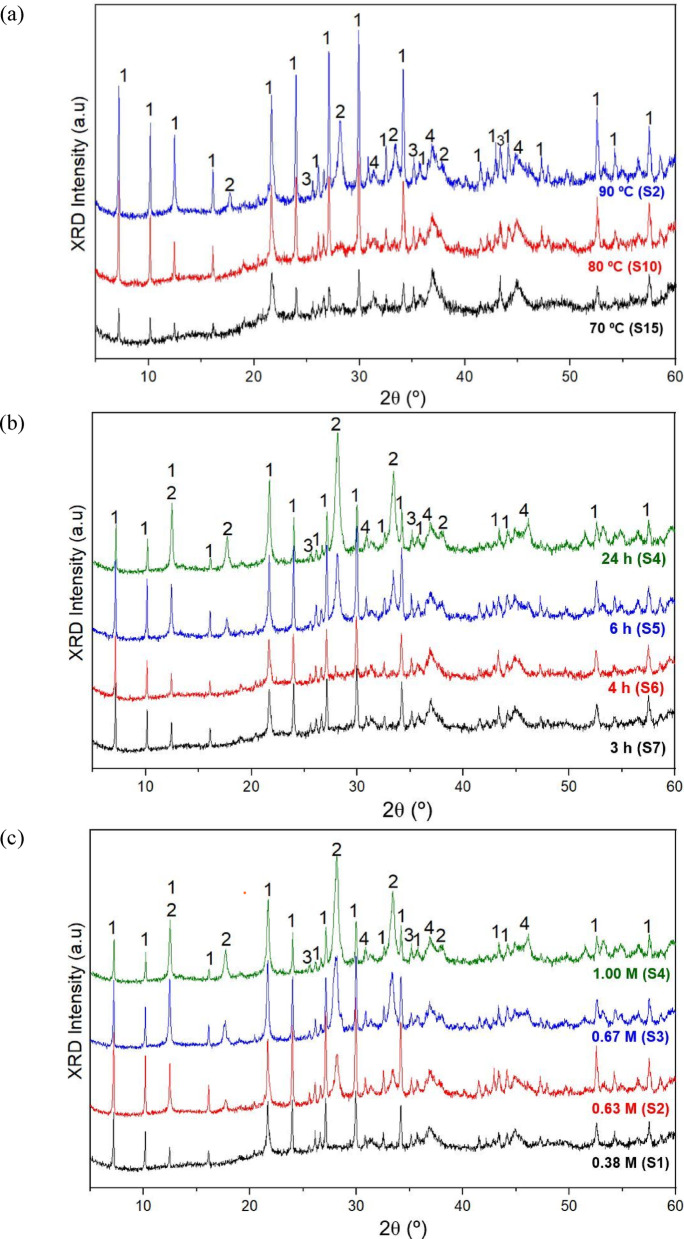
XRD patterns of zeolites synthesised under different hydrothermal conditions. (**a**) 24 h using a 0.63 M NaOH solution at temperatures ranging from 70–90 °C; (**b**) at 90 °C using 1 M NaOH solution for different synthesis times; and (**c**) at 90 °C for 24 h using different NaOH concentrations. Identified crystalline phases: (1) LTA zeolite, (2) NaP zeolite, (3) corundum, and (4) spinel.

Diatomite sludge (DS) was supplied by the brewery Estrella de Levante, S.A.U., located in Murcia, Spain. This waste was dried at 90 °C to eliminate the water content. The dried solid was then calcined at 540 °C for 4 h to obtained calcined diatomite sludge (CDS) and to remove organic matter (mainly yeast), which could otherwise cause unpleasant odours and bacterial growth. In its dried form, DS is a fine brownish powder, whereas after calcination the CDS becomes white to yellowish in colour, with a particle size < 160 μm. The chemical composition of CDS consists mainly of silica (89.8 wt%), together with 3.1 wt% Al_2_O_3_ and minor metal oxides. Mineralogically, CDS is a highly amorphous, although small amounts of quartz and cristobalite can be detected by XRD (Fig. 1S). This amorphous structure favours the incorporation of silicon into the zeolitic framework^45^ and allows the use of mild synthesis conditions, such as lower alkali concentration and shorter reaction times, as extensive dissolution of stable crystalline phases is not required.

As an alkalising agent, a sodium hydroxide solution was employed, prepared by dissolving NaOH pellets (98% purity, technical grade, Panreac) in distilled water at different concentrations.

### One-pot zeolite synthesis

Zeolite synthesis was performed using a conventional hydrothermal method, without any prior activation of the raw materials and in the absence of organic structure-directing agents. In a typical experiment, predetermined amounts of aluminium salt slag (AlSS) and calcined diatomite sludge (CDS) were first combined to achieve a fixed molar SiO₂/Al₂O₃ ratio of 1. The solid mixture was then transferred to a 1 L Teflon-lined stainless-steel autoclave (Parr Instruments). Subsequently, an aqueous sodium hydroxide solution (0.38–1 M) was added as the alkalising agent to form a reaction slurry. The autoclave was sealed, and the synthesis was conducted under autogenous pressure with continuous stirring at temperatures between 70 and 90 °C for reaction times ranging from 2 to 24 h. Upon completion of the hydrothermal treatment, the autoclave was cooled to room temperature, and the resulting solid product was recovered by filtration. The solid was thoroughly washed with distilled water until neutral pH and finally dried at 100 °C for 24 h prior to further characterisation.

### Characterisation techniques

The chemical composition of the wastes was determined by X-ray fluorescence (XRF) using a Bruker S8 Tiger spectrometer on pressed pellets.

The mineralogical composition of the wastes and synthesised materials was analysed by X-ray diffraction (XRD) using an X’ Pert MPD diffractometer equipped with CuKα radiation. Data were collected over a scanning range of 5° ≤ 2θ ≤ 60° at a scanning speed of 0.5° min^−1^. Phase identification and semi-quantitative analysis of zeolites in the products were performed using DIFFRAC.Suite EVA Plus 13.0 software (Bruker, AXS GmbH, Karlsruhe, Germany), which employs the Powder Diffraction File (PDF) database and is based on the Reference Intensity Ratio (RIR, I/Ic) method. After background subtraction and phase identification by search-match, the experimental diffraction patterns were compared with reference patterns (PDF files 73–2340 and 89–6322 for LTA and NaP zeolites, respectively). Scale factors were calculated from the relative integrated intensities of the identified crystalline phases and converted into weight percentages using the corresponding RIR values, with optional correction for mass absorption coefficients when applicable. The crystallite size (D) of the synthesised zeolites was estimated using the Scherrer equation: D = (0.9·λ)/(FWHM·cos θ), where λ is the X-ray wavelength (0.154 nm), FWHM is the full width at half maximum (in radians), and θ is the Bragg diffraction angle (in radians) corresponding to the most intense diffraction peak.

Textural characterisation of the zeolites was carried out by nitrogen adsorption/desorption measurements at 77 K using a Micromeritics ASAP 2010 instrument. Prior to analysis, the samples were degassed at 150 °C under vacuum for 24 h. The specific surface area (S_BET_) was determined via the BET method in the P/P_0_ range of 0.002–0.3 using a 14-point linear fit. The external surface area (S_EXT_), defined as the area of pores other than micropores (mesopores, macropores and the outer surface of the particle), and the pore volume were calculated using the t-plot method from the slope of the 19-point linear fit in the thickness (t) range of 0.22–0.74 nm, according to the Harkins–Jura equation. The pore size distribution was determined using the Barrett–Joyner–Halenda (BJH) method from the adsorption branch of the isotherms.

Fourier transform infrared (FTIR) spectra were recorded using KBr pellets in the range of 400–4000 cm^− 1^ with a Thermo Scientific Nicolet 6700 spectrophotometer.

The cation exchange capacity (CEC) of the zeolites was determined using the standardised ammonium ion-exchange method with a 1 M NH_4_Cl solution^46^.

The morphology of the zeolites was examined by field-emission scanning electron microscopy (FESEM) using a Hitachi S-4800 microscope on powder samples coated with a thin layer of graphite.

## Results and discussion

The experimental conditions employed in the different tests carried out to obtain zeolites from the AlSS and CDS are summarised in Table 1, together with the relative content in the final products.

**Table 1:** Experimental conditions for the synthesis of zeolite from AlSS and CDS and the relative zeolite content.

Sample	T (ºC)	t (h)	[NaOH] (M)	Zeolite content (%)
S1	90	24	0.38	56.9
S2		24	0.63	73.2
S3		24	0.67	82.4
S4		24	1	86.5
S5		6		83.0
S6		4		62.8
S7		3		62.0
S8		2		41.3
S9	80	24	0.50	49.5
S10		24	0.63	54.9
S11		6	0.63	41.3
S12		6	1	60.6
S13		4		64.1
S14		2		38.9
S15	70	24	0.63	47.3

The XRD patterns of all synthesised materials are shown in Fig. 1, illustrating the evolution of the crystalline phases as a function of the main experimental parameters affecting zeolite formation, such as temperature (Fig. 1a), reaction time (Fig. 1b) and alkali concentration (Fig. 1c). Hydroxyl ions play a fundamental role in the breaking and rebuilding of Al‒Si bonds during the condensation and rearrangement reactions that lead to the formation of prenuclei and, subsequently, to particle growth. In addition, both synthesis time and temperature strongly influence whether the final product consists predominantly LTA or NaP zeolite. The diffraction patterns indicate that, after hydrothermal treatment, LTA and NaP zeolites are formed as the major phases, whereas corundum and spinel —originating from unreacted AlSS components‒ are detected as minor phases.

Figure 1a shows a progressive increase in the intensity of the peaks associated with LTA zeolite as the synthesis temperature increases. However, higher temperatures also favour the formation of additional phases, leading to the appearance of NaP zeolite at 90 °C. This phase transformation is consistent with Ostwald’s rule^47^, which states that the least thermodynamically stable polymorph forms first and is subsequently replaced by more stable phases. Such successive transformations are very common in zeolite chemistry, making the controlled formation of single–phase, highly crystalline LTA zeolite within the LTA/NaP system particularly challenging and worthy of detailed investigation. Furthermore, the overall crystallinity of the synthesised materials increased gradually with temperature. Kim and Ahn^48^ reported that reaction temperature has a strong influence on zeolite nucleation and crystal growth, as higher temperatures supply more energy to the system, reduce the time required for phase formation, and favour the development of more crystalline products. The interconversion between zeolite phases has also been reported by Burriesci et al.^49,]^ who observed a transformation between zeolite A and sodalite as the temperature varied between 70 and 95 °C. They concluded that the interconversion reflects a degree of metastability between the two zeolite structures under identical reaction conditions.

Figure 1b illustrates the evolution of the zeolite phases as the synthesis time increased from 3 to 24 h. After 3 h, well–developed diffraction peaks corresponding to LTA zeolite were observed. At 4 h, low–intensity peaks attributable to NaP zeolite appeared in addition to those of LTA. At 6 h, the peaks of both LTA and NaP became more intense and well defined. After 24 h, the intensity of LTA peaks decreased slightly, whereas the NaP peaks became more pronounced, indicating a decrease in LTA/NaP ratio over time due to the transformation of LTA into NaP. This transformation suggests a partial collapse of the cubic lattice characteristic of LTA zeolite, leading to the formation of crystals with the tetragonal lattice typical of NaP zeolite. Accordingly, the peak at 2θ = 12.5°, matches the [1 1 0] plane common to both structures, increases in intensity primarily due to the growth of the NaP phase. Meftah et al.^50^ reported similar changes in zeolite phases over time, with halloysite as the main precursor. Akın et al.^51^ also described the synthesis of 4 A zeolite from fly ash at temperatures between 75 and 100 °C and crystallisation times ranging from 2.5 to 7 h, depending on the SiO_2_/Al_2_O_3_ ratio and the aging method used prior to synthesis. In the present study, an increase in the crystallinity of the sample is observed from 3 to 6 h, mainly due to the high development of the LTA zeolite, whereas the total crystallinity remained nearly constant between 6 and 24 h. Residual crystalline phases such as corundum and spinel remained undissolved throughout the process and were retained in the final product without significantly affecting zeolite formation.

The diffractograms in Fig. 1c show a progressive increase in the NaP zeolite content with increasing NaOH concentration. A single-phase LTA product was obtained at 0.38 M NaOH, whereas mixtures of LTA and NaP were formed at NaOH concentrations ≥ 0.63 M. For all concentrations tested, the highest zeolite content was achieved at 1 M NaOH (Table 1). Increasing alkalinity enhances waste dissolution, generating higher concentrations of silicate and aluminate species in the reaction medium and favouring zeolite formation. Under these conditions, complete dissolution of AlSS and CDS is promoted, resulting predominantly in NaP zeolite formation. The development of LTA zeolite is governed by the dissolution rate of the precursors, the number and distribution of nuclei in the initial seeds, and kinetics of crystal growth during hydrothermal treatment. These factors are influenced by other variables, such as the synthesis temperature, crystallisation time, initial composition, and molar ratios, among others parameters^32^.

The results demonstrate that zeolite content is strongly dependent on temperature, NaOH concentration, and reaction time (Table 1). At 90 °C, zeolite content increased significantly with alkalinity, reaching 86.5% at 1 M NaOH (S4), compared with 56.9% at 0.38 M (S1). Reaction time also had a pronounced effect, with zeolite content increasing from 41.3% after 2 h (S8) to 83.0% after 6 h (S5) under otherwise identical conditions. Similarly, increasing temperature enhanced zeolite formation, with samples synthesised for 24 h at 0.63 M NaOH exhibiting yields of 47.3%, 54.9%, and 73.2% at 70 °C (S15), 80 °C (S10), and 90 °C (S2), respectively. These trends confirm that higher alkalinity, longer reaction times, and elevated temperatures favour aluminosilicate dissolution and nucleation, thereby accelerating zeolite crystallisation and crystal growth.

**Fig. 2:**
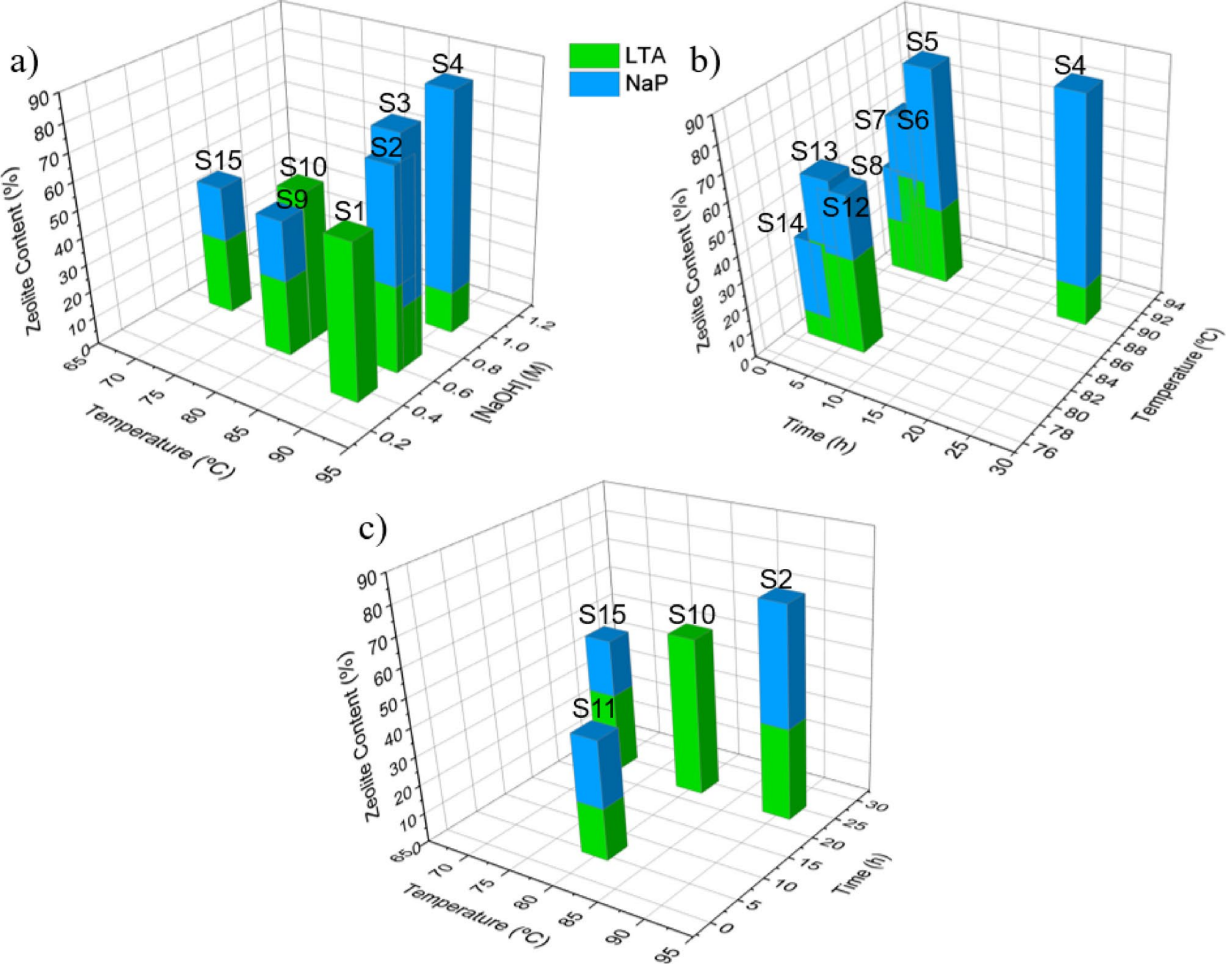
Relative contents of LTA and NaP zeolites as a function of synthesis parameters. (**a**) Effect of temperature and NaOH concentration after 24 h. (**b**) Effect of time and temperature at a fixed NaOH concentration of 1 M. (**c**) Effect of time and temperature at a fixed NaOH concentration of 0.63 M.

Crystallite sizes were determined from the most intense diffraction peaks of both zeolites, namely the peak centred at 2θ ~ 29.9° for LTA and the peak located at approximately 28.1° for NaP (Fig. 2S, Table 1S). The crystallite size of LTA ranged between 49 and 61 nm, whereas that of NaP varied between 19 and 23 nm, being smaller than that of LTA zeolites. These values are in good agreement with previously reported studies, in which the growth of LTA and NaP zeolite crystals synthesised from rice husk ash and aluminium salt slag was distinguished, with crystallite sizes in the ranges of 45–53 nm and 21–28 nm, respectively^20,21^. Among the synthesis parameters investigated, crystallite size was most strongly influenced by NaOH concentration, with D values in the range of 50–60 nm for the LTA zeolite. No significant variations in the crystallite size of NaP zeolite were observed under the different experimental conditions studied.

Figure 2 shows the relative contents of LTA and NaP zeolite obtained under different synthesis conditions, highlighting their strong influence on phase selectivity. Higher NaP contents were favoured by longer reaction times, higher temperatures and NaOH concentrations. In contrast, LTA zeolite was obtained as a single zeolitic phase only in samples S1 and S10. Accordingly, the LTA/NaP ratio could be tuned by adjusting the synthesis parameters. Thus, the LTA zeolite content varies from 10% (S14) to 56.9% (S1), whereas the NaP content varies from 20% (S8) to 72% (S4).

**Fig. 3:**
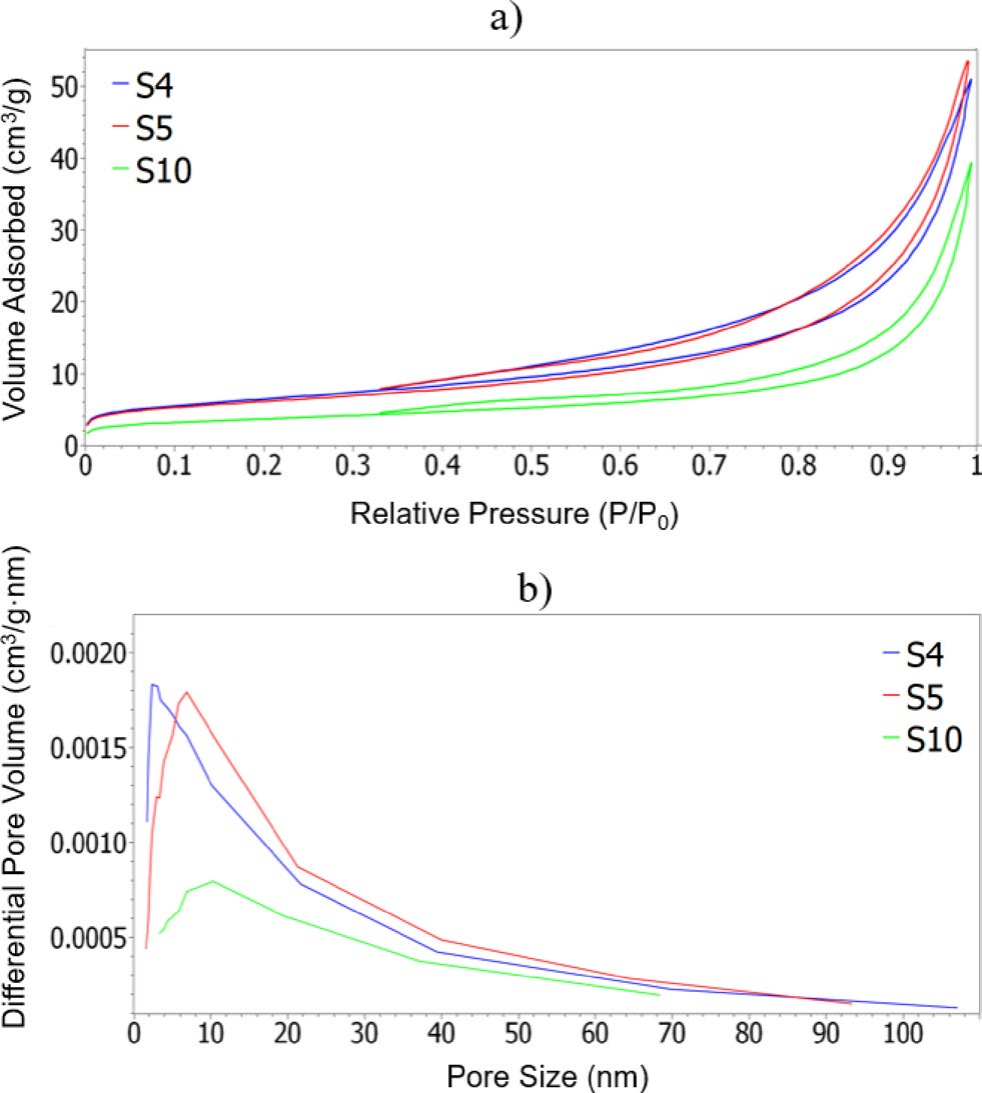
Nitrogen adsorption‒desorption isotherms (**a**) and pore size distributions (**b**) of the three waste–derived zeolites.

The FTIR spectra of the CDS and of the samples with the highest NaP (S4) and LTA (S10) contents are shown in Fig. 3S. The absence of CDS characteristic bands in S4 indicates complete reaction of the silica source during hydrothermal synthesis. Conversely, FTIR bands at 1100, 794 and 471 cm^− 1^, are still observed in S10, suggesting incomplete CDS conversion under milder synthesis conditions.

The textural properties of the waste-derived zeolites were analysed for samples with the highest NaP (S4) and LTA (S10) content and for sample with a mixture of both LTA and NaP zeolites (S5). Figure 3 shows the nitrogen adsorption‒desorption isotherms (Fig. 3a) and pore size distributions (Fig. 3b). At intermediate relative pressures, the desorption branch does not coincide with the adsorption branch, resulting in a hysteresis loop associated with capillary condensation and indicating the presence of mesopores in the zeolite structure. According to the International Union of Pure and Applied Chemistry (IUPAC) classification, all the samples exhibited type IV isotherms (Fig. 3a). In all cases, an H3-type hysteresis loop is observed over the P/P_0_ range of 0.35–0.98, which is characteristic of materials containing slit-shaped pores with non-uniform sizes. Pore size distributions were evaluated using the Barrett–Joyner–Halenda (BJH) method (Fig. 3b), and the resulting textural parameters, including S_BET_ and S_EXT_ are summarised in Table 2. All the samples display combined micro- and mesoporosity, with pore size predominantly in the range of 2–10 nm, depending on the dominated zeolitic phase. LTA-containing samples exhibit larger pore sizes than those dominated by NaP zeolite. A clear increase in BET surface area with increasing total zeolite content is clearly observed. Furthermore, although S4 and S5 show similar overall zeolite contents, the sample with the highest NaP content (S4) exhibits the highest S_BET_ value. The relatively low pore volumes measured for all samples are likely related to the bottleneck geometry of the micropores, which restricts gas diffusion and leads to an underestimation of pore volume^52^. The specific surface areas obtained are comparable to those reported for NaP zeolites obtained from aluminium slag and rice husk ash, with reported S_BET_ values of 17.5–22.1 m^2^ g^−1^[20]. Similarly, NaP zeolite synthesised from the same aluminium slag exhibited an S_BET_ of 14.2 m^2^ g^−1^[44], while LTA zeolite prepared from fine aluminium slag collected by sleeve filters showed an S_BET_ of 19.7 m^2^ g^− 1^[43].

Table 2 also reports the cation exchange capacity (CEC) values. Overall, CEC increases with increasing total zeolite content. For samples with comparable zeolite contents (S4 and S5, both exceeding 80%), the higher proportion of NaP zeolite in S4 (72%) results in a higher CEC value. These findings are consistent with previous studies reporting CEC values of 2.73 and 2.86 meq g^−1^ for NaP zeolites prepared from aluminium dross^20,44^. For comparison, a commercial LTA zeolite was also analysed, exhibiting a CEC of 2.11 meq g^−1^, which is of the same order of magnitude as the values obtained in this study.

**Table 2:** Textural properties and CEC of the synthesised waste-derived zeolites.

Sample	S_BET_ (m^2^ g^− 1^)	S_Ext_ (m^2^ g^− 1^)	Pore volume (cm^3^ g^− 1^)	Pore size (nm)	CEC (meq g^− 1^)
S4	22.5	21.4	0.079	2.4	2.54 ± 0.08
S5	21.2	19.2	0.083	6.8	1.87 ± 0.04
S10	12.7	11.4	0.061	10.5	1.57 ± 0.07

Table 3 summarizes the textural and ion-exchange properties of the zeolite synthesized in this work (S4) and compares them with those of waste-derived zeolites previously reported in the literature, obtained through different raw materials and synthesis strategies. Although the textural and ion-exchange properties of sample S4 are comparable to those reported for zeolites synthesized from industrial wastes, S4 uniquely combines competitive performance with a one-pot synthesis route, direct use of two industrial wastes from different sectors, mild operating conditions, and the absence of secondary solid waste generation. This combination highlights the novelty and practical relevance of the proposed approach.

**Table 3:** Comparative textural and ion-exchange properties of waste-derived zeolites reported in the literature and synthesized in this work.

Sample	Process	Raw materials	T (ºC)/t (h)	S_BET_ (m^2^ g^−1^)	CEC (meq g^−1 ^)	Key remarks
S4 (this work)	One-pot	Salt slag + Diatomite sludge	90/24	22.5	2.5	Direct use of two wastes Cross-sector industrial synergy No secondary solid waste Mild conditions
NaP^20^	Alkaline extraction of Si/Al	Salt slag + Rice husk ash	105/20	21.1	3.7	Pre-extraction step required
NaP^44^	One-pot (with reagents)	Al dust + Na_2_SiO_3(technical)_	120/6	14.2	2.7	Technical reagents required
NaP^53^	One-pot	Fly ash	125–150/8–24	–	2.0–2.7	High temperature
NaP^54^	Magnetic separation	Fly ash	100–150/24	–	1.7–3.7	High temperature
LTA^39,43^	One-pot (with reagents)	Al dust + Na_2_SiO_3(technical)_	80/12	19.7	1.7	Technical reagents required
LTA^55^	Multi-step	Kaolin	60/4 + 90/3.5	13.4	–	Raw material is not a waste
LTA^34^	Multi-step	Biomass fly ash	90/12	13.7	–	Secondary residues

**Fig. 4:**
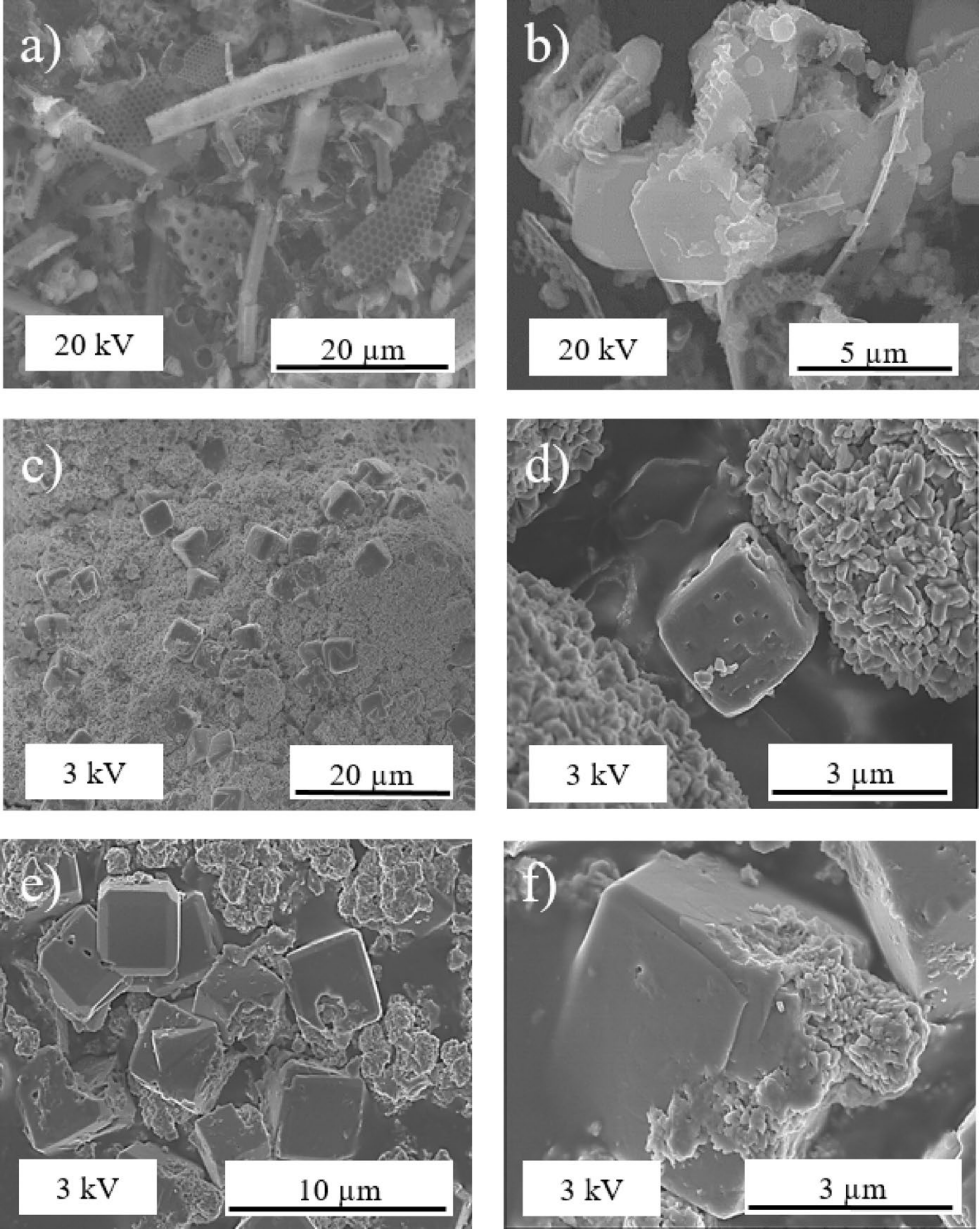
Field-emission scanning electron microscopy (FESEM) micrographs of (**a**) calcined diatomite sludge (CDS). (**b**) Sample S10. (**c**, **d**) Sample S4 at different magnifications. (**e**, **f**) Sample S5 at different magnifications.

The microstructures of CDS and samples S4, S5 and S10 are shown in Fig. 4. CDS exhibits a porous microstructure composed of particles with different sizes and shapes, typical of diatoms frustules^56^(Fig. 4a). Owing to the mechanical handling during beer filtration and subsequent drying, the diatom structures appear fragmented rather than fully preserved. Sample S10, obtained under mild conditions (80 °C and 0.63 M NaOH), shows the characteristic morphology of LTA zeolite, consisting of cube-shaped crystals^57^ (Fig. 4b). In addition to LTA crystals, unreacted diatom fragments are observed between the cubes, in agreement with the FTIR results. The microstructure of sample S4 (Fig. 4c and d) reveals the coexistence of LTA and NaP phases. Small cube-shaped crystals of LTA with sizes of 3.4–3.6 μm are clearly identified, together with larger crystallite aggregates exhibiting a cauliflower-like morphology characteristic of a NaP structure^20^, which is consistent with the corresponding XRD results. Finally, the FESEM images of sample S5 (Fig. 4e and f) show a higher LTA content and a lower NaP content compared with sample S4. Cubic LTA crystals with an average size of approximately 3 μm and bevelled edges are observed. This relatively rather atypical shape of the LTA zeolite crystals was explained by computational studies, which linked this shape to the characteristic lattice structure of LTA zeolite^58^. This lattice consists of truncated octahedral cages, called β-cages or sodalite cages, connected by double 4-membered rings (D4Rs). The combination of these cages and D4R creates large empty spaces, called α-cages, which are connected by 8-membered rings (8R). Čejka et al.^59^ also reported that the most stable orientation of LTA zeolite is [1 0 0] plane, which ends in complete sodalite cages and corresponds to a surface energy of 0.09 J m^−2^. In contrast, the [1 1 1] plane, which terminates in D4Rs, exhibits a higher surface energy of 0.15 J m^−2^. The greater stability of the [1 0 0] orientation explains the tendency of LTA crystals to adopt a cubic morphology at equilibrium. Notably, Fig. 4f captures the progressive transformation of LTA into NaP zeolite, evidenced by the transition from the less stable cubic lattice of LTA to the more stable tetragonal lattice of NaP. This transformation occurs without prior amorphization of the LTA crystals, indicating a topotactic transformation between the two zeolite structures.

## Conclusions

This study demonstrates the technical feasibility of synthesising zeolites from industrial wastes, specifically aluminium salt slag and diatomite sludge, used as unconventional sources of aluminium and silicon, respectively. The chemical and mineralogical characteristics of these wastes make them suitable precursors for the production of zeolitic materials.

Hydrothermal synthesis resulted in the formation of LTA and NaP zeolites, with their relative proportions strongly governed by synthesis parameters such as temperature, reaction time, and NaOH concentration. Mild conditions favoured the formation of single-phase LTA, whereas increasing these parameters promoted a topotactic transformation towards the thermodynamically more stable NaP phase.

The resulting crystallite sizes ranged from 48 to 61 nm for LTA and from 18 to 23 nm for NaP. The crystallite size of LTA was mainly influenced by NaOH concentration, while synthesis time and temperature had a limited effect. The specific surface area (S_BET_) varied between 12.7 and 22.5 m² g^−1^, with higher values associated with NaP-rich samples, whereas larger pore sizes were observed in LTA-dominant materials. Cation exchange capacity (CEC) values reached up to 2.54 meq g^−1^, particularly for NaP-enriched compositions, and were comparable to those of natural and other waste-derived zeolites. These properties highlight the potential of the as-obtained waste-derived zeolites as effective adsorbents or ion exchangers for environmental applications.

Considering the strategic importance of zeolites and the scale of their global market, together with the substantial generation of aluminium salt slag and its significant aluminium content, the valorisation of this hazardous waste as a feedstock for zeolite synthesis represents an effective recycling pathway. Moreover, this work exemplifies industrial symbiosis by integrating two waste streams from distinct sectors into a single value-added process, thereby conserving natural resources and reducing waste management demands. The proposed approach supports circular economy principles and highlights the potential of co-recycling strategies for advancing more sustainable material cycles.

## Supplementary Information

Below is the link to the electronic supplementary material.

Supplementary Material 1

## Data Availability

The datasets used and/or analysed during the current study are available from the corresponding author on reasonable request.
