# Supplementary material for: Harnessing salt slag and diatomite sludge by co-recycling for zeolite production
Source: Sci Rep. 2026 Apr 24;16:19039. doi: 10.1038/s41598-026-50164-3 (PMC13279793; doi:10.1038/s41598-026-50164-3)
Supplement: Supplementary file 1 — Supplementary Material 1 [file 41598_2026_50164_MOESM1_ESM.docx]

**Harnessing salt slag and diatomite sludge by corecycling for zeolite production**

Rafael Carrizosa, Isabel Padilla, Maximina Romero and Aurora López-Delgado*

MEDES Group, Materials Department, Eduardo Torroja Institute for Construction Sciences, IETcc-CSIC. C/ Serrano Galvache, 4, 28033 Madrid, Spain.

*Corresponding author email: [alopezdelgado@ietcc.csic.es](mailto:alopezdelgado@ietcc.csic.es) (Aurora López-Delgado)

Figure 1S shows the XRD patterns of DS and CDS. Both diffractograms are characteristic of highly amorphous materials, showing only three broad peaks corresponding to the quartz and cristobalite phases. This amorphous structure favours the incorporation of silicon into the zeolitic lattice^1^, and allows the use of mild synthesis conditions (lower concentration of alkali agent and shorter reaction times) since it is not necessary to dissolve large amounts of stable crystalline structures. The calcination of DS does not promote the formation of new crystalline phases and CDS shows an XRD pattern identical to that of DS, without increasing the intensity of the peaks associated with the crystalline phases.


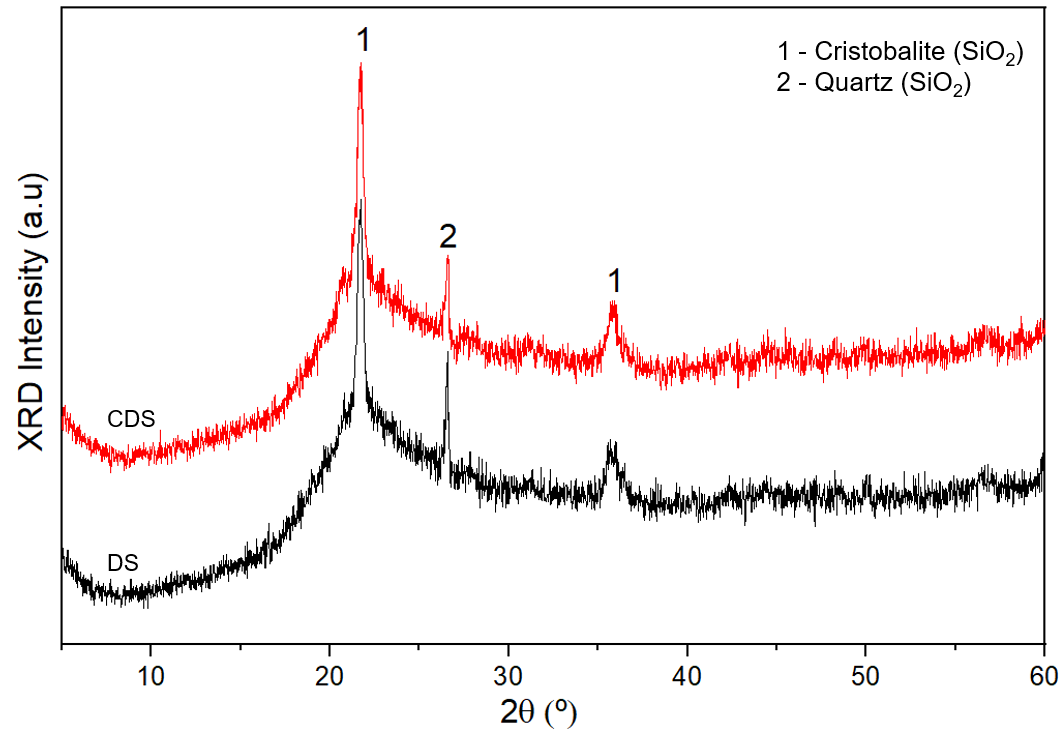


**Fig. 1S.** XRD pattern of DS (black) and CDS (red).

To determine the crystallite size, the LTA peak centred at 2θ ~ 29.9º ([h k l] = [6 4 4]) and the NaP peak located at approximately 28.1º ([h k l] = [-1 3 1]) were selected. Figure 2S depicts the variation in the profile of these peaks with experimental synthesis conditions, and Table 1S collects the values of 2θ, intensity, FWHM, and crystallite size (D) for LTA and NaP. The crystallite size for the synthesised LTA zeolites is in the range of 49 - 61 nm interval, while for the NaP zeolites it varies in the range of 19 - 23 nm, which is smaller than that of the LTA zeolites. These results are in good agreement with other studies, where authors were able to distinguish the growth of LTA and NaP zeolite crystals from rice husk ash and ASS, with crystallite sizes ranging between the 45 - 53 nm and 21 - 28 nm, respectively^2,3^. Among the synthesis parameters studied, the crystallite size is most influenced by the NaOH concentration, with D values in the range of 50 - 60 nm for LTA zeolite. No significant variations in the crystallite size are observed for the NaP zeolite with respect to the experimental conditions.


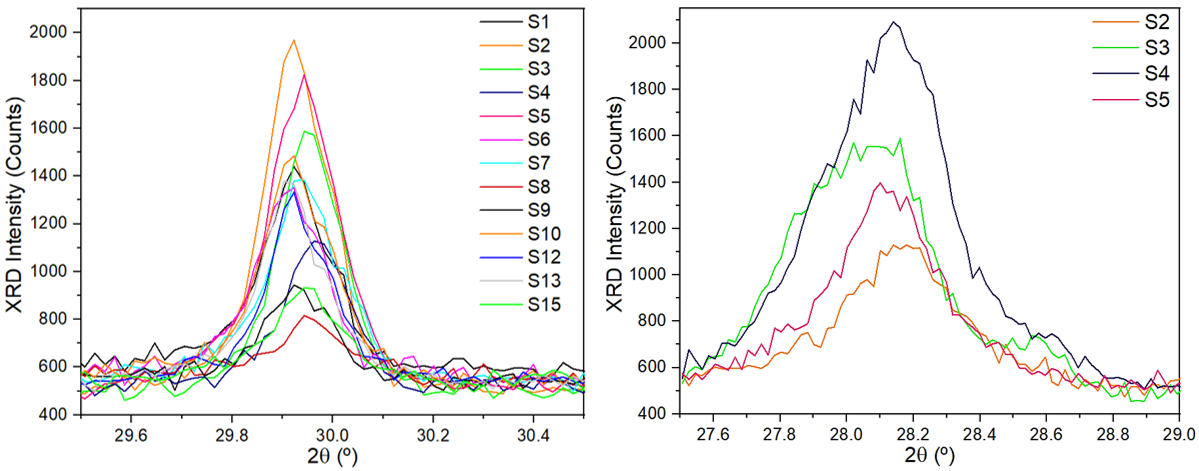
**Fig. 2S.** Variation of the [6 4 4] peak of LTA (left) and [-1 3 1] peak of NaP (right) zeolites.

| Zeolite Type | Sample | 2θ (º) | Intensity (counts) | FWHM (º) | D (nm) |
| --- | --- | --- | --- | --- | --- |
| LTA | S1 | 29.92 | 1431 | 0.164 | 50.1 |
|  | S2 | 29.92 | 1951 | 0.158 | 52.0 |
|  | S3 | 29.95 | 1596 | 0.146 | 56.3 |
|  | S4 | 29.96 | 1134 | 0.138 | 59.5 |
|  | S5 | 29.94 | 1819 | 0.155 | 53.0 |
|  | S6 | 29.92 | 1342 | 0.165 | 49.8 |
|  | S7 | 29.94 | 1394 | 0.162 | 50.8 |
|  | S8 | 29.94 | 810 | 0.136 | 60.5 |
|  | S9 | 29.92 | 937 | 0.162 | 50.8 |
|  | S10 | 29.92 | 1485 | 0.161 | 51.1 |
|  | S12 | 29.92 | 1320 | 0.144 | 57.1 |
|  | S13 | 29.90 | 1377 | 0.150 | 54.8 |
| NaP | S2 | 28.17 | 1125 | 0.375 | 21.8 |
|  | S3 | 28.16 | 1581 | 0.457 | 17.9 |
|  | S4 | 28.14 | 2096 | 0.426 | 19.2 |
|  | S5 | 28.11 | 1401 | 0.358 | 22.9 |

**Table 1S** Values of 2θ, intensity, FWHM of the [6 4 4] peak for LTA and of the [-1 3 1] peak for NaP, and calculated crystallite sizes (D).

Figure 3S shows the FTIR spectra of CDS and the samples with the highest NaP (S4) and LTA (S10) contents. For the calcined diatomite sludge, the stronger band at 1100 cm^-1^ can be attributed to the Si-O-Si asymmetric stretching vibration of crystalline SiO_2_. The band at 794 cm^-1^ is assigned to the symmetric stretching vibration of the individual SiO_4_ tetrahedra in the structure of the α-cristobalite framework. The band at 471 cm^-1^ is due to the Si-O-Si bending^1,5^. In samples S4 and S10, the asymmetric Al-O stretching is located in the 1100-1000 cm^-1^ region, while the symmetric Al-O stretching is located in the 794 cm^-1^ region. The bands at 687 and 471 cm^-1^ belong to the internal vibrations of Al or Si in tetrahedral TO_4_ positions (T = Si or Al). Finally, the band at 554 cm^-1^ is related to the vibrations of the 8-membered double rings of the LTA and NaP zeolites^5,6^. The characterisation of the zeolites by FTIR is supported by mineralogical characterisation by XRD, since mixtures of LTA/NaP zeolites often show similar infrared bands, making it difficult to distinguish between them on the basis of FTIR analysis alone. It should be noted that the characteristic bands in the CDS spectrum are not present in S4, indicating that all the CDS have reacted to form zeolite during the hydrothermal treatment. Conversely, in sample S10, the contributions of the FTIR peaks of CDS are observed at 1100, 794 and 471 cm^-1^, indicating that the reaction of CDS was not complete due to the mild experimental conditions and, accordingly some fraction of CDS could remain in the final product.


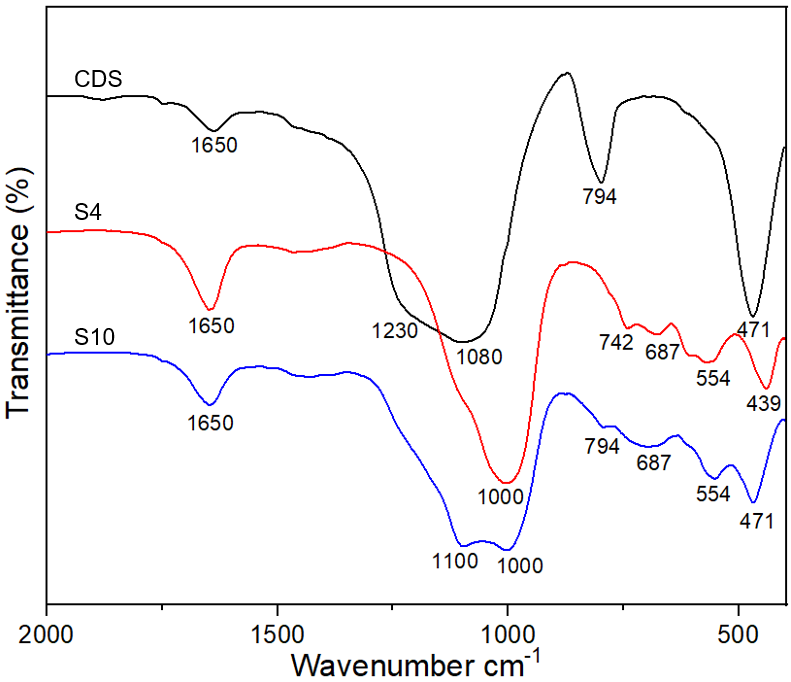


**Figure 5:** Zeolites LTA and NaP produced with variation of temperature and [NaOH] at constant time of 24 h (a), changing time and temperature setting a constant [NaOH] of 1 M (b) and [NaOH] of 0.63 M (c).

**Fig. 3S.** FTIR spectra of CDS, and samples S4 and S10.

**References**

1. Sheng, G., Dong, H. & Li, Y. Characterization of diatomite and its application for the retention of radiocobalt: role of environmental parameters. *J. Environ. Radioact.* 113, 108–115. <https://doi.org/10.1016/j.jenvrad.2012.05.011> (2012).
2. Ritter, M. T., Lobo-Recio, M. Á., Padilla, I., Romero, M. & López-Delgado, A. Salt slag and rice husk ash as raw materials in zeolite synthesis: process optimization using central composite rotational design. *Sustain. Chem. Pharm.* 39, 101599. <https://doi.org/10.1016/j.scp.2024.101599> (2024).
3. Ritter, M. T., Padilla, I., Lobo-Recio, M. Á., Romero, M. & López-Delgado, A. Waste symbiosis through the synthesis of highly crystalline LTA and SOD zeolites. *Materials* 17, 4310. <https://doi.org/10.3390/ma17174310> (2024).
4. Wang, W., Yin, H., Jiang, N., Jin, G. & Wang, Z. Using brewing waste diatomite activated by ball milling to simple and sustainable synthesis of nano-H-ZSM-5 aggregates. *J. Environ. Chem. Eng.* 12, 112239. <https://doi.org/10.1016/j.jece.2024.112239> (2024).
5. Ríos, C. A., Williams, C. D. & Fullen, M. A. Nucleation and growth history of zeolite LTA synthesized from kaolinite by two different methods. *Appl. Clay Sci.* 42, 446–454. <https://doi.org/10.1016/j.clay.2008.05.006> (2009).
6. Sharma, P., Song, J. S., Han, M. H. & Cho, C. H. GIS-NaP1 zeolite microspheres as potential water adsorption material: influence of initial silica concentration on adsorptive and physical/topological properties. *Sci. Rep.* 6, 22734. <https://doi.org/10.1038/srep22734> (2016).
